# Reduced Opioid Use Among Patients Who Received Liposomal Bupivacaine Brachial Plexus Block for Total Shoulder Arthroplasty

**DOI:** 10.7759/cureus.55516

**Published:** 2024-03-04

**Authors:** Paulina Szakiel, Nicholas Aksu, Maxwell D Gruber, Kyle Zittel, Brandon Stryder, Evan Argintar

**Affiliations:** 1 Orthopaedics, Georgetown School of Medicine, Washington, USA; 2 Surgery, Washington State University (WSU) Elson S. Floyd College of Medicine, Spokane, USA; 3 Orthopaedics, MedStar Georgetown University Hospital, Washington, USA; 4 Orthopaedics, MedStar Washington Hospital Center, Washington, USA

**Keywords:** in-hospital length of stay, opioid, reverse total shoulder arthroplasty, total shoulder arthroplasty, liposomal bupivacaine

## Abstract

Purpose

This retrospective cohort explores the efficacy of regional shoulder blocks using Exparel™ in patients undergoing total shoulder arthroplasty (TSA)/reverse total shoulder arthroplasty (RSA) to reduce total opioid prescription, refills, and length of stay in the acute care setting.

Methods

Patients who underwent TSA/RSA by a single surgeon in a three-year period were evaluated. Patients in the case group received liposomal bupivacaine 1.3% brachial plexus block while the control group received ropivacaine 0.5% interscalene brachial plexus block. Outcomes of the study included the number of opioids taken, opioids prescribed, and length of hospital stay.

Results

Thirty-six patients underwent TSA/RSA between January 2017 and March 2020. Patients who received an Exparel brachial plexus block had decreased opioid use within the first 24 hours after surgery compared to the ropivacaine group, 9.00 ± 14.10 and 26.20 ± 24.8 morphine milligram equivalent (MME), respectively (p=0.0213). Patients who received an Exparel brachial plexus block had decreased opioid prescriptions over the entire postoperative follow-up, 411.00 ± 200.74 MME in the case group and 593.07 ± 297.57 MME in the control group (p=0.0314). Lastly, patients who received an Exparel brachial plexus block had a shorter length of hospital stay, 1.28 ± 0.91 days as compared to the control group's 2.15 ± 1.49 days (p=0.0451).

Conclusion

This study demonstrates a significant reduction in opioid prescribing and use in patients who receive Exparel brachial plexus nerve blocks compared to non-liposomal local anesthetics, as well as a significant reduction in the length of hospital stay. The data suggest that Exparel use may decrease the risks associated with opioid use while providing adequate analgesia in patients undergoing shoulder arthroplasty.

## Introduction

In recent years, total shoulder arthroplasty (TSA) and reverse shoulder arthroplasty (RSA) have become two of the most widely adopted surgical intervention procedures for chronic shoulder pathology [1]. As such, optimizing postoperative management is a paramount goal to minimize postoperative discomfort and optimize function [2-4]. A juxtaposed balance between effective pain management and the side effects of narcotics, including sedation, constipation, and nausea, creates a dilemma for both patient and physician. More specifically, over-prescription of opioids has been shown to be the number one risk factor for prescribed opioid-induced overdose in the US [5,6]. As a result, medical professionals have sought different modalities of pain management, including the use of preoperative brachial plexus nerve block (NB). However, a recent systematic review of NB in shoulder surgery only showed an advantage in opioid prescribing at postoperative day (POD) 0, with an increase in requirement at POD 7 compared to patients without NB [7]. The current anesthesiology evidence review highlights a lack of clear protocols for post-TSA pain management [8].

In the use of local anesthetics, lipid solubility influences the duration of action [9]. Exparel™ (Pacira Pharmaceuticals, Inc., Parsippany, NJ, USA), an extended-release liposomal bupivacaine equivalent, is a Food and Drug Administration (FDA)-approved medical formulation intended for intraoperative use to reduce the necessity of postoperative pain medications [10]. Its liposomal formulation provides extended postoperative analgesia, with a half-life ranging between 9 and 34 hours depending on the procedure location [11]. Recurrent randomized control trials (RCTs) have shown Exparel™ to be an effective addition to postoperative pain management [12,13]. In 2018, Exparel™ received FDA approval for use as a nerve block for regional anesthesia in shoulder surgery. The clinical trial supported reduced opioid use and postoperative pain scores in those who used Exparel compared to placebo (normal saline) [14]. Additionally, local liposomal bupivacane has been shown to be equivalent to non-liposomal nerve block when comparing postoperative opioid use while reducing the length of hospital stay [15,16]. However, a comparison of an Exparel brachial plexus nerve block to a non-liposomal anesthetic brachial plexus block in reducing opioid use following TSA is lacking. Consequently, the purpose of this retrospective cohort comparison is to explore the efficacy of Exparel nerve blocks compared to standard non-liposomal formulations of anesthetics in patients undergoing TSA. We will evaluate efficacy by the ability of Exparel to reduce total opioid prescriptions, refills, and overall length of stay in the acute care setting.

This article was previously presented at the Virginia Orthopedic Society Annual Meeting on April 29, 2023.

## Materials and methods

Study population

In this retrospective cohort comparison, current procedural terminology (CPT) code 23474 was identified for the evaluation and analysis of all patients who had undergone primary TSA/RSA at a single orthopedic practice. IRB approval was granted by Georgetown University School of Medicine (STUDY00003485) prior to the initiation of the study. The start and end points between January 2017 and March 2020 were clearly established. Patients who had undergone TSA/RSA between 01/01/2017 and 03/01/2020 were included. Exclusion criteria included the following: patients who were prescribed opioids less than 90 days before surgery, patients who received a left and right TSA in the same week as well as a revised TSA, and patients with incomplete and/or missing electronic medical record data. The electronic medical records were retrospectively reviewed by two independent reviewers (P.M.S. and B.S.) and were evaluated for demographic data. This data included age, sex, BMI, date of procedure, date of discharge, liposomal bupivacaine use, opioid prescription on the day of surgery, and total number of opioids prescribed. The surgical history/report was further explored to evaluate for additional factors. These factors included past surgical procedures, the types of procedures, and any recorded surgical complications. Finally, postoperative clinic notes were reviewed and evaluated for any information relating to opioid prescriptions, repeat pain measurements, and refills.

Surgical technique

Preoperatively, the anesthesiologist completed the brachial plexus nerve block. The patient was placed in a supine captain’s chair position. A deltopectoral incision was made, the bicep tendon was identified, and soft tissue tenodesis was performed. The subscapularis muscle was reflected off of the lesser tuberosity. The humeral head was cut in the anatomic version. The glenoid was then exposed, reamed, and prepared using the standard technique, and a polyethylene glenoid (for TSA) or baseplate (for RSA) component was placed. The proximal humerus implant was placed, and the shoulder was reduced. For anatomic TSA, the subscapularis muscle was repaired to the lesser tuberosity using the transosseous technique with two Arthrex (Naples, FL) swivel locks and fiber tape. For RSA, no subscapularis repair was performed. Finally, the primary incisional fascia and skin were closed.

Pain management protocol

All patients underwent a preoperative ultrasound-guided interscalene brachial plexus nerve block performed by a board-certified anesthesiologist. Patients in the case group received 1.3% liposomal bupivacaine as the local anesthetic while patients in the control group received 0.5% ropivacaine. Liposomal bupivacaine use began in February 2018 and was used alongside non-liposomal anesthetic treatment for the remainder of the study. Postoperative care included a standard prescription of an opioid (including morphine, oxycodone, hydromorphone, or fentanyl), Celebrex, and Tylenol to be taken as needed for pain based on the patient's reported pain score. Patients in the case group were informed that they would receive liposomal bupivacaine as their anesthetic for the nerve block.

Statistical analysis

The primary outcome of this study was the quantity of opioids prescribed after the procedure and the sum of opioids prescribed during all subsequent follow-up visits. These values were obtained from electronic health record (EHR) prescription records and converted to oral morphine milligram equivalent (MME) for standardization. Secondary outcomes included the amount of PRN opioids given within the first 24 hours after surgery and the total length of hospital stay.

Statistical analysis was performed using JMP Pro software, version 14 (SAS Institute, Inc., Cary, North Carolina, USA). Patient demographics were compared via a Fisher exact test for categorical variables and a 2-sample t-test for continuous variables. A 2-sample F test was used to determine the variance between groups, followed by a 2-tailed unequal variance t-test. Statistical significance was assigned at P<0.05.

## Results

A total of 36 patients were included in the study, with 14 in the case group and 22 in the control group. The case group had a mean age of 67.50 ± 10.39 years, and the control group had a mean age of 69.68 ± 10.82 years. The case group had 9 (64%) females and 5 males, and the control group had 20 (91%) females and 2 males. The case group included 8 TSA (57%) and 6 RSA, and the control group included 12 TSA (55%) and 10 RSA. There was no significant difference in patient demographics between the two groups, including age (p = 0.5536), gender (p = 0.0842), and percent TSA vs rTSA (p = 1.000).

Postoperative opioid prescriptions

**Figure 1:**
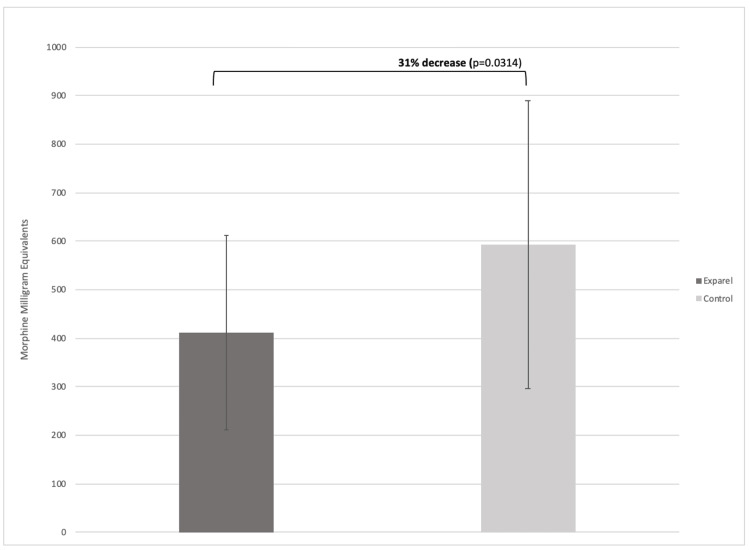
Mean number of morphine milligram equivalents prescribed to patients after shoulder arthroplasty

The quantity of opioids prescribed was converted to oral MME according to Centers for Disease Control and Prevention guidelines. The total quantity of opioids prescribed over the entire course of postoperative follow-up in the Exparel group was 411.00 ± 200.74 MME and 593.07 ± 297.57 MME in the control group (p=0.0314) (Figure 1).

**Figure 2:**
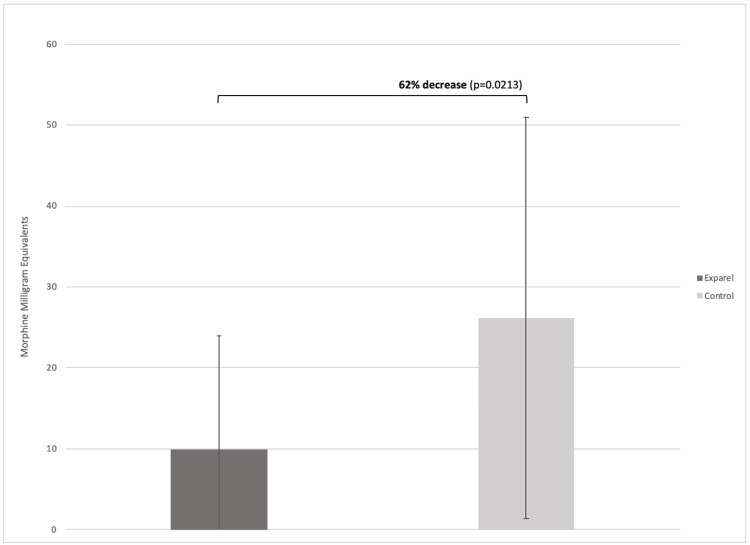
Mean number of morphine milligram equivalents given to patients within 24 hours after shoulder arthroplasty

Mean quantities of MME prescribed to the Exparel and control groups within the first 24 hours after surgery were 9.00 ± 14.10 MME and 26.20 ± 24.8 MME, respectively (p=0.0213) (Figure 2).

**Figure 3:**
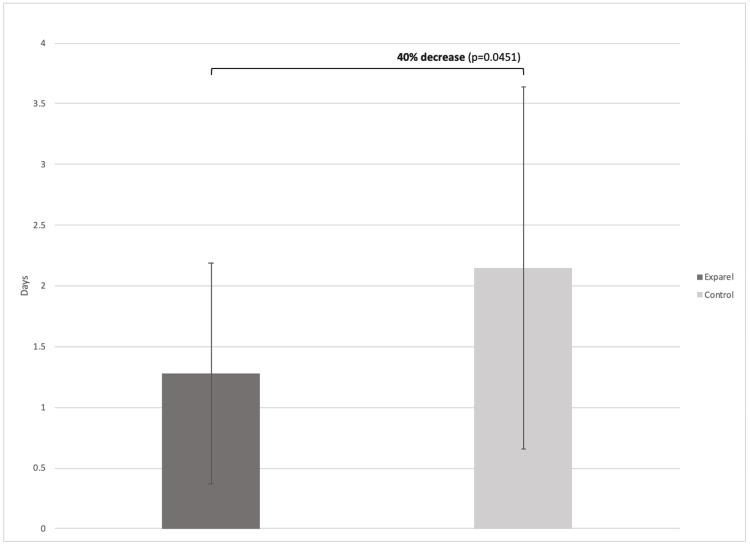
Mean length of stay of patients after shoulder arthroplasty

The length of hospital stay was significantly lower in the Exparel group, with a mean hospital stay of 1.28 ± 0.91 days, and 2.15 ± 1.49 days in the control group (p=0.0451) (Figure 3).

## Discussion

Orthopedic procedures remain a large contributor to opioid prescribing, as studies have shown patients receiving up to three times the opioids following upper extremity procedures [17]. The dangers of opioid addiction are widely recognized. However, multiple studies have linked overdose mortality to the availability of opioids, even when correctly prescribed [18]. This highlights the need for opioid-reducing techniques in pain management following orthopedic procedures. As such, the American Pain Society (APS) recommends an amalgamated treatment - utilizing a multimodality approach [2]. A conservative pain regimen for those undergoing TSA includes conservative means (non-steroidal anti-inflammatory drugs (NSAIDs), ice, heat, compression, and temporary stasis of movement). As pain management progresses, further modalities are explored. These include a combination of local anesthetics and/or oral/intravenous (IV) steroids [2]. Given the current cultural paradigm and consensus on opioids, opioid prescription to patients can be difficult to navigate. Recent studies have shown no significant difference in pain score between TSA and reverse TSA (rTSA) as soon as four hours postoperatively, enabling the combined comparison utilized in this study [19].

The current study found a significant decrease of 32% in total opioid prescribing following TSA in the group receiving a liposomal bupivacaine nerve block (p = 0.0314). These data are comparable to findings from analyses of other orthopedic procedures, including anterior cruciate ligament (ACL) reconstruction and total knee arthroplasty (TKA), which showed significantly reduced postoperative opioid use with utilization of intraoperative liposomal bupivacaine [20,21]. This finding differs from a previous randomized trial by Harttrup et al., which found no difference in post-op opioid use at the three-day and three-week time points. Data in this study showed a trend toward reduced MMEs in the LB group; however, significance was not found [22].

Analysis of TSA and RSA with a pain management protocol, including liposomal bupivacaine, showed a 62% decrease in opioid consumption within 24 hours following surgery compared to traditional pain management methods (p = 0.0213). This reduction of 17.2 MME may be clinically significant with the Centers for Disease Control and Prevention (CDC) suggesting 20-50 MME/day having some risk of the adverse effects of opioid use [23]. These results are consistent with a previous randomized control trial showing reduced postoperative opioid use in multiple procedures, including bunionectomy, total knee arthroplasty, hemorrhoidectomy, and submuscular augmentation mammoplasty, following the inclusion of liposomal bupivacaine in pain management [10]. Additionally, these results support a randomized control trial comparing ultrasound-guided brachial plexus block with liposomal bupivacaine to standard pain management following shoulder surgery, including TSA. The study found significantly reduced opioid consumption, up to 48 hours following surgery, in the group receiving liposomal bupivacaine treatment [24]. The liposomal encapsulation of the drug allows the medication to be delivered over a longer duration of time, resulting in longer-acting efficacy and an improved side-effect profile [25].

Finally, this study found a significantly shorter hospital stay afforded for the liposomal bupivacaine treatment group, with an average reduction of 0.87 days (p = 0.0451). The effect found in this study was larger than was found in a previous meta-analysis comparing liposomal bupivacaine to interscalene nerve block, which found an average of 0.16 days reduced stay in the liposomal bupivacaine treatment group [16]. This reduction in hospital stay can have a significant effect on patient health and satisfaction following surgery, as longer hospital stays are associated with increased morbidity and lower patient satisfaction [26].

Limitations

The study has the following limitations: The sample size included only 14 out of 36 total patients, a result of only a single senior surgeon utilizing this technique during the study period. This resulted in a study power of 72.01%. Additionally, the number of opioids prescribed can include unused medication and may overestimate the amount prescribed to a patient. Additionally, the number of opioids initially prescribed to the case group could act as a confounding variable in this study. The low initial dose could create bias by influencing patient expectations regarding future opioid supply. Finally, the overall length of stay in the case group may also be attributed to changes in hospital protocols after 2018 and an overall decrease in the length of stay for all surgeries.

## Conclusions

As one of the highest physician prescribers of opioids in the United States, orthopedic surgeons must utilize opioid-sparing techniques. This study demonstrates a significant reduction in opioid prescribing and length of hospital stay in patients who receive Exparel brachial plexus nerve blocks compared to standard non-liposomal local anesthetics. The data suggest that Exparel use may significantly decrease the risks associated with opioid use while providing adequate analgesia in patients undergoing shoulder arthroplasty. Additional investigation into the optimal pain control protocol after TSA is needed.
